# Impact of the COVID-19 pandemic on emergent stroke care in Beijing, China

**DOI:** 10.1038/s41598-023-31530-x

**Published:** 2023-03-17

**Authors:** Yuan Wang, Gang Liu, Yu Zhu, Haiqing Song, Yi Ren, Ying Liu, Qingfeng Ma

**Affiliations:** 1 Department of Neurology, Xuanwu Hospital, Capital Medicine University, Beijing, 100053 China; 2 Beijing Stroke Quality Control Centre, Beijing, 100053 China; 3 Medical Administration and Management Office, Beijing Municipal Commission of Health, Beijing, 100053 China

**Keywords:** Cerebrovascular disorders, Stroke, Therapeutics

## Abstract

The coronavirus disease 2019 (COVID-19) pandemic has caused an unprecedented disruption to health care systems around the globe. Stroke is still an ongoing issue during the pandemic. We investigated the impact of the COVID-19 outbreak on emergent stroke care in Beijing, China. This is a retrospective analysis of two groups of patients with acute ischaemic stroke (AIS) registered in the Beijing Emergency Care Database between January 1, 2019, and December 31, 2020. Based on a database including 77 stroke centres, the quantity and quality of emergency care for stroke were compared. Subgroup analyses based on hospitals in different areas (high-risk and low/medium-risk areas) were carried out. A total of 6440 and 8699 admissions with suspected stroke were recorded in 2020 and 2019, respectively. There were no significant differences in the mean age and sex distribution for the patients between the two observational periods. The number of AIS admissions decreased by approximately 23.9% during the COVID-19 pandemic compared to that during the prepandemic period. The proportions of intravenous thrombolysis and endovascular treatment were 76.4% and 13.1%, respectively, in 2020, which were higher than those in 2019 (71.7% and 9.3%, respectively). There was no statistically significant difference in the time from stroke onset to arrival at the hospital (97.97 ± 23.09 min vs. 99.40 ± 20.76 min, *p* = 0.832) between the two periods. The door-to-needle time for thrombolysis (44.92 ± 9.20 min vs. 42.37 ± 9.06 min, *p* < 0.001) and door-to-thrombectomy time (138.56 ± 32.45 min vs. 120.55 ± 32.68 min, *p* < 0.001) were increased significantly in the pandemic period compared to those in the prepandemic period, especially in hospitals in high-risk areas. The decline in the number of patients with AIS and delay in treatment started after the launch of the level-1 public health emergency response and returned to stability after the release of professional protocols and consensus statements. Disruptions to medical services during the COVID-19 pandemic have substantially impacted AIS patients, with a clear drop in admission and a decline in the quality of emergent AIS care, especially in hospitals in high-risk areas and at the time of the initial outbreak of COVID-19. Health care systems need to maintain rapid adaptation to possible outbreaks of COVID-19 or similar crises in the future.

## Introduction

Severe acute respiratory syndrome coronavirus type 2 (SARS-CoV-2) is the agent of coronavirus disease 2019 (COVID-19), which was first diagnosed on December 8, 2019, in a patient in Wuhan, China^1^. The outbreak of the novel coronavirus pneumonia spread rapidly across the country, and many provinces and municipalities launched a major public health emergency response to control the pandemic. Although the COVID-19 pandemic in China was brought under control to some extent by the end of the Wuhan shutdown on April 8, 2020, the virus still spread rapidly around the rest of the world, and it is too early for us to ease off. As the Public Health Emergency of International Concern declared by the WHO, official data showed more than 88,269,340 diagnosed cases all over the world, including 1,794,726 deaths up to December 31, 2020.

Ischaemic stroke is still a major cause of disability and death worldwide. To reduce mortality and disability, hyperacute care involving reperfusion treatments, acute stroke unit management and subsequent rehabilitation must be applied in patients with acute ischaemic stroke (AIS)^2,3^. Intravenous rt-PA has been widely recommended as a standard treatment for AIS in most clinical practice guidelines^4^. Mechanical thrombectomy has been a significant advancement for the treatment of patients with stroke related to large vessel occlusions^5^. However, the application of rt-PA thrombolysis or endovascular intervention in clinical settings is restricted by the narrow therapeutic time window. Time plays a predominant role in revascularization. Previous studies have found that a favourable functional outcome was more frequently achieved in the early time window, and the proportion of patients with favourable outcomes decreased with an increased time gap between stroke onset and treatment initiation^6–9^. Striving for a better prognosis and racing against the clock to shorten the treatment time is of paramount importance^10^.

During the pandemic, the management of patients with AIS could be challenging, and certain precautions must be taken to protect health care workers and patients. A negative impact on acute stroke care has been reported by several studies, and the effect of the pandemic on stroke care is not only in quantity but also in quality^10–12^. Due to the local infection rate of COVID-19, the local regulations to contain the pandemic, the public attitude towards the pandemic, etc., the impact of COVID-19 on stroke services in different regions is likely to differ.

Based on data from the Beijing Emergency Care Database, we compared the evaluation and treatment process of AIS patients who entered stroke emergency management before and during the pandemic, with the aim of evaluating the impact of the pandemic on emergent care for AIS patients. The results may offer valuable information to reorganize or optimize the current strategies of emergency care and to better prepare for future challenging times.

## Methods

### Study design and participants

This retrospective study assessed the impact of the SARS2-Cov-2 outbreak on the process of emergency care for AIS patients. We consecutively included all patients with suspected symptoms of AIS entering stroke emergency management between January 1, 2020, and December 30, 2020, as the pandemic group. Similar patients with the same time span between January 1, 2019, and December 30, 2019, were included as the prepandemic group. All consecutive adult patients ≥ 18 years of age were eligible for inclusion. The diagnosis of AIS was made by physicians based on positive neuroimaging results.

### Data collection

Data were obtained from the Beijing Emergency Care Database, an integrated database that connects the emergency medical services (EMS) system and emergency departments (EDs) in secondary and tertiary hospitals in Beijing. For acute stroke, patients from the 77 hospitals eligible for AIS acute care, included in the First Aid Treatment Map for Stroke (FATMS) published by Beijing Municipal Health Commission, were all recorded by a standard platform^13^. This smartphone platform named “Green” was developed to standardize and streamline the overall AIS emergency management processes and strengthen prehospital and in-hospital coordination^14^. Since January 2018, it has been freely available to the EMS systems and all eligible hospitals for AIS therapy in Beijing. Since then, the Beijing Emergency Care Database has been updated in real time. In the database, all emergency admissions for stroke were recorded, with a group of key metrics measuring the quality of acute stroke care, including time records for last known well and hospital arrival, images, intravenous thrombolysis (IVT), endovascular treatment (EVT), revascularization, and so on^12^.

Paramedics, physicians, and nurses in all stroke centres eligible for AIS emergency management, as well as hospital management and quality control groups in Beijing, were trained several times to use this platform^14^. For patients with suspected AIS who were transported by the ambulance, the paramedics initiated the patient’s records on the platform during transportation. For patients who arrived at the hospitals by themselves, doctors who received the suspected AIS patient would activate and complete clinic records for patients on the platform. The records were then automatically entered into the electronic clinic records in the chosen hospitals. Data records were not mandatory. However, the data integrity, validity and timeliness were assessed by the Beijing Municipal Health Commission, which would influence the ranking of the AIS first aid performance of each hospital and ED.

Using the time records for the key therapy points, outside quality control parties can compare and analyse the data and adjust the qualification of AIS first aid facilities. Quality control groups inside hospitals can use the information to find weak points during therapy courses. The Beijing Municipal Health Commission and other quality control parties released quality control reports every month to all stroke first aid facilities^14^.

In this study, the number of hospital admissions, the number of AIS patients, and several quality measures for acute care of ischaemic stroke, such as onset to door (OTD) time, door to CT scan (DTC) time, door-to-needle (DTN) time, door to puncture (DTP) time, onset to needle (OTN) time, and onset to recanalization (OTR) time, were obtained from the database. The eligibility for IVT and EVT was in accordance with recent guidelines.

To explore the correlation between stroke emergency hospital admissions and the number of newly diagnosed COVID-19 cases, the monthly number of COVID-19 cases in Beijing was obtained from the Beijing Municipal Health Commission.

**Figure 1:**
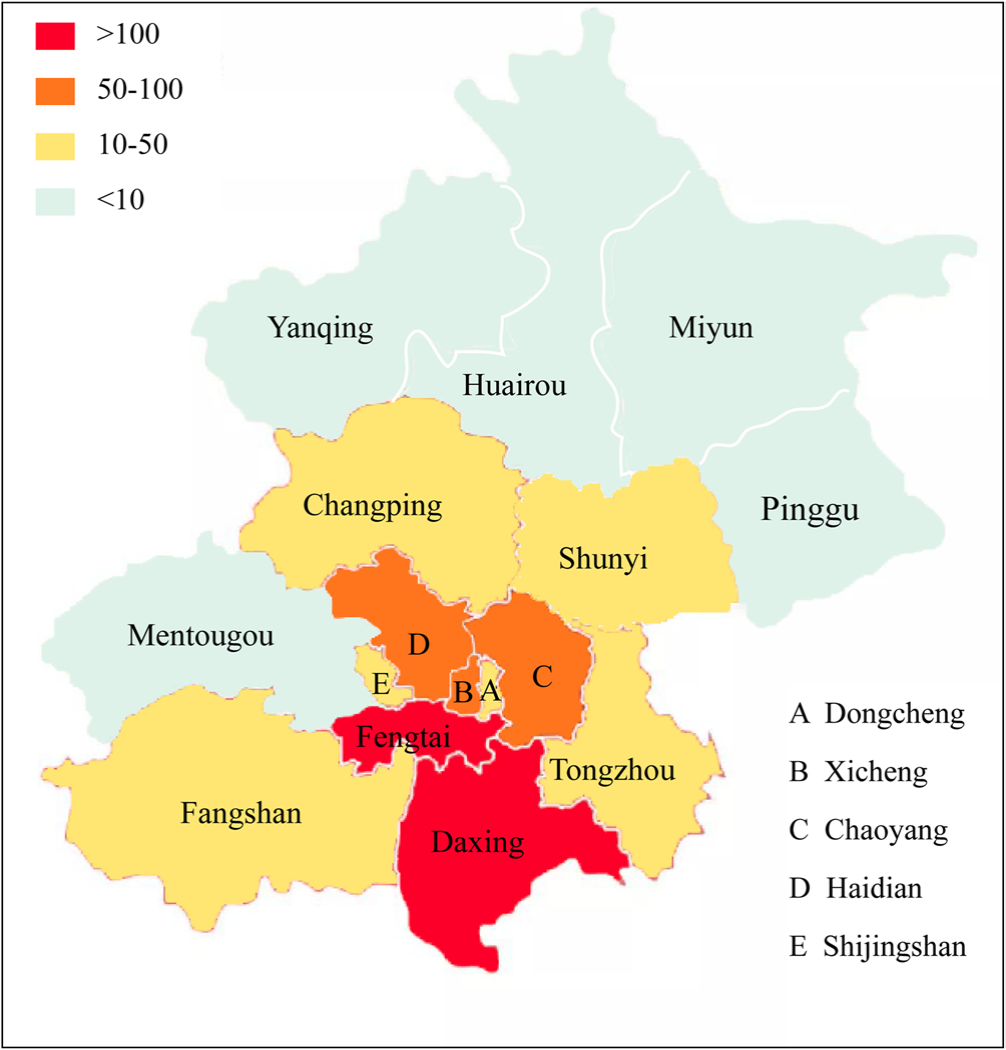
Map of the 16 districts in Beijing. The different colors represent the accumulative numbers of local diagnosed COVID-19 cases in 2020.

There are 16 districts in Beijing (Fig. 1). We divided these districts into 2 areas depending on the accumulative number of locally diagnosed COVID-19 cases in 2020. Compared with other regions of China, such as Wuhan and Guangzhou, there was a relatively lower infection rate of COVID-19 in Beijing during 2020, in which no more than 1000 COVID-19 cases occurred. Areas with high risk were defined as those with total cases in 2020 exceeding 50, including Fengtai, Daxing, Haidian, Chaoyang, and Xicheng. Areas with medium or low risk (fewer than 50 cases) included Dongcheng, Shijingshan, Tongzhou, Shunyi, Fangshan, Changping, Pinggu, Huairou, Mentougou, Yanqing, and Miyun.

### Statistical analysis

SPSS statistical software, version 27.0 (SPSS Institute, Inc., Chicago, IL, USA), was used for all statistical analyses. We performed two-tailed t tests for normally distributed continuous variables and chi-squared tests for confirmatory variables. A Mann‒Whitney U test was performed in cases in which the variable was not normally distributed. *P* values below 0.05 were considered statistically significant.

### Ethics considerations

The study was reviewed and approved by the Ethics Committee of Xuanwu Hospital, Capital Medical University (No. [2021]053). The study was conducted in accordance with the principles of the Declaration of Helsinki. The data are anonymous, and written informed consent for participation was not required for this study in accordance with the national legislation and institutional requirements^15^.

## Results

### Number of hospital admissions

In the database, there were 6440 and 8699 admissions with suspected stroke recorded during the pandemic period in 2020 and the counterpart in 2019, respectively, which revealed a reduction of 26.4% in 2020 compared to 2019. No significant differences were found in the mean age and sex distribution for the patients between the two observational periods. The reduction in hospitals in the high-risk areas (27.6%) was slightly higher than that in the low/medium-risk areas (23.1%). A greater reduction was observed in haemorrhagic stroke compared with ischaemic stroke (49.2% vs. 23.9%, *p* < 0.001). In 2020, 50.7% of the patients arrived at hospitals by emergency medical services, while 43.8% of the patients arrived by private cars. Compared with those in 2019, the reduction in emergency hospital admissions transferred by private cars was higher than that transferred by ambulance (30.9% vs. 21.0%, *p* < 0.001). After deleting 650 and 1089 patients in each period with a diagnosis of other diseases or with an unknown diagnosis, the remaining 5790 and 7610 stroke emergency admissions were included for analysis (Table 1).

**Table 1:** Effects of the pandemic on the number of admissions in Beijing.

	2019	2020	Reductions (%)
All admissions with suspected stroke, n	8699	6440	26.4
Male, n (%)	5880 (67.6)	4456 (69.2)	–
Age (mean ± SD)	65.03 ± 12.39	66.54 ± 16.36	–
Arrival route^a^, n			
Emergency medical services, n	4266	3371	30.9
Private vehicle, n	4091	2826	21.0
In-hospital stroke, n	337	239	29.1
COVID-19 cases in the local district, n			
< 50 (medium or low-risk area)	3203	2463	27.6
≥ 50 (high-risk area)	5496	3977	23.1
Stroke types^a^, n			
Ischemic stroke	7610	5790	23.9
Haemorrhagic stroke	767	390	49.2

^a^The difference in distributions between two periods was statistically significant (*P* < 0.05).

### Quality metrics for AIS

A reduction of 19.0% was observed in the patients treated with IVT in 2020 compared with that in 2019 (4423 vs. 5460). The numbers of EVTs in 2020 and 2019 were 756 and 709, respectively. The percentages of the patients undergoing IVT and EVT were 76.4% and 13.1%, respectively, in 2020 and 71.7% and 9.3%, respectively, in 2019. The proportions of patients receiving IVT or EVT therapy were higher in 2020 than in 2019 (Table 2).

**Table 2:** Quality measures of the acute care provided for ischemic stroke between 2020 and 2019.

	All hospitals		*P*	Hospitals in the high-risk area		*P*	Hospitals in the low/medium-risk area		*P*
	2019	2020		2019	2020		2019	2020	
Type of therapy for acute ischemic stroke, n			< 0.001			< 0.001			0.02
Intravenous thrombolysis	5460	4423		3215	2430		2245	1993	
Endovascular treatment	709	756		501	523		208	233	
Onset to door (OTD), min	99.40 ± 20.76	97.97 ± 23.09	0.832	113.00 ± 20.31	116.00 ± 22.36	0.830	93.23 ± 18.62	89.45 ± 18.91	0.642
Door to CT (DTC), min	16.88 ± 2.96	18.44 ± 3.81	0.205	15.6 ± 2.88	17.0 ± 3.46	0.507	17.45 ± 2.94	19.09 ± 3.94	0.283
Door-to-needle (DTN), min	42.37 ± 9.06	44.92 ± 9.20	< 0.001	38.94 ± 6.67	42.40 ± 6.46	< 0.001	47.30 ± 9.74	47.99 ± 10.95	0.029
Onset to needle (OTN), min	144.72 ± 9.72	145.33 ± 11.53	0.005	144.26 ± 5.58	148.59 ± 5.94	< 0.001	145.39 ± 13.54	135.33 ± 11.99	0.864
Door to puncture (DTP), min	120.55 ± 32.68	138.56 ± 32.45	< 0.001	123.62 ± 23.29	147.39 ± 25.71	< 0.001	113.16 ± 47.59	118.75 ± 37.02	0.167
Onset to recanalization (OTR), min	405.61 ± 60.65	422.55 ± 98.84	< 0.001	419.26 ± 50.81	448.86 ± 82.47	< 0.001	372.73 ± 69.33	363.50 ± 106.91	0.278

**Figure 2:**
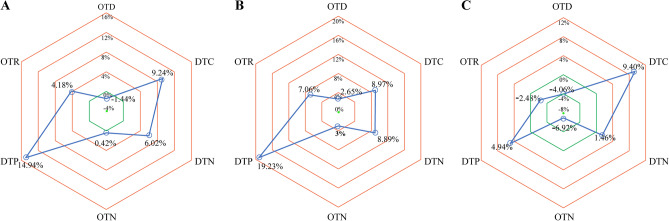
The ratios of changes of mean times from 2019 to 2020. (**A**) The ratios of changes of mean times from 2019 to 2020 in all hospitals. (**B**) The ratios of changes of mean times from 2019 to 2020 in hospitals in the high-risk areas. (**C**) The ratios of changes of mean times from 2019 to 2020 in hospitals in the low/medium-risk areas.

The time in workflow for AIS is presented in Table 2. For the prehospital stage, there was no significant difference in the OTD times between the two groups. However, the OTD time in 2020 was shorter than that in 2019 in hospitals in low/medium-risk areas, without significance. After hospital arrival, the patients admitted during the COVID-19 period had no significant delay in DTC compared with the patients admitted in the pre-COVID-19 period. The DTN, OTN, DTP and OTR times in 2020 were significantly longer than those in 2019 (Fig. 2). In hospitals in the high-risk areas, there was a statistically significant delay in the DTN, OTC, DTP and OTR times during the COVID-19 versus pre-COVID-19 periods. In the whole emergency care workflow, there was no significant difference in the ONT, DNP and ORT times between the two periods, except for the longer DTN time in the hospitals in low/medium-risk areas.

**Figure 3:**
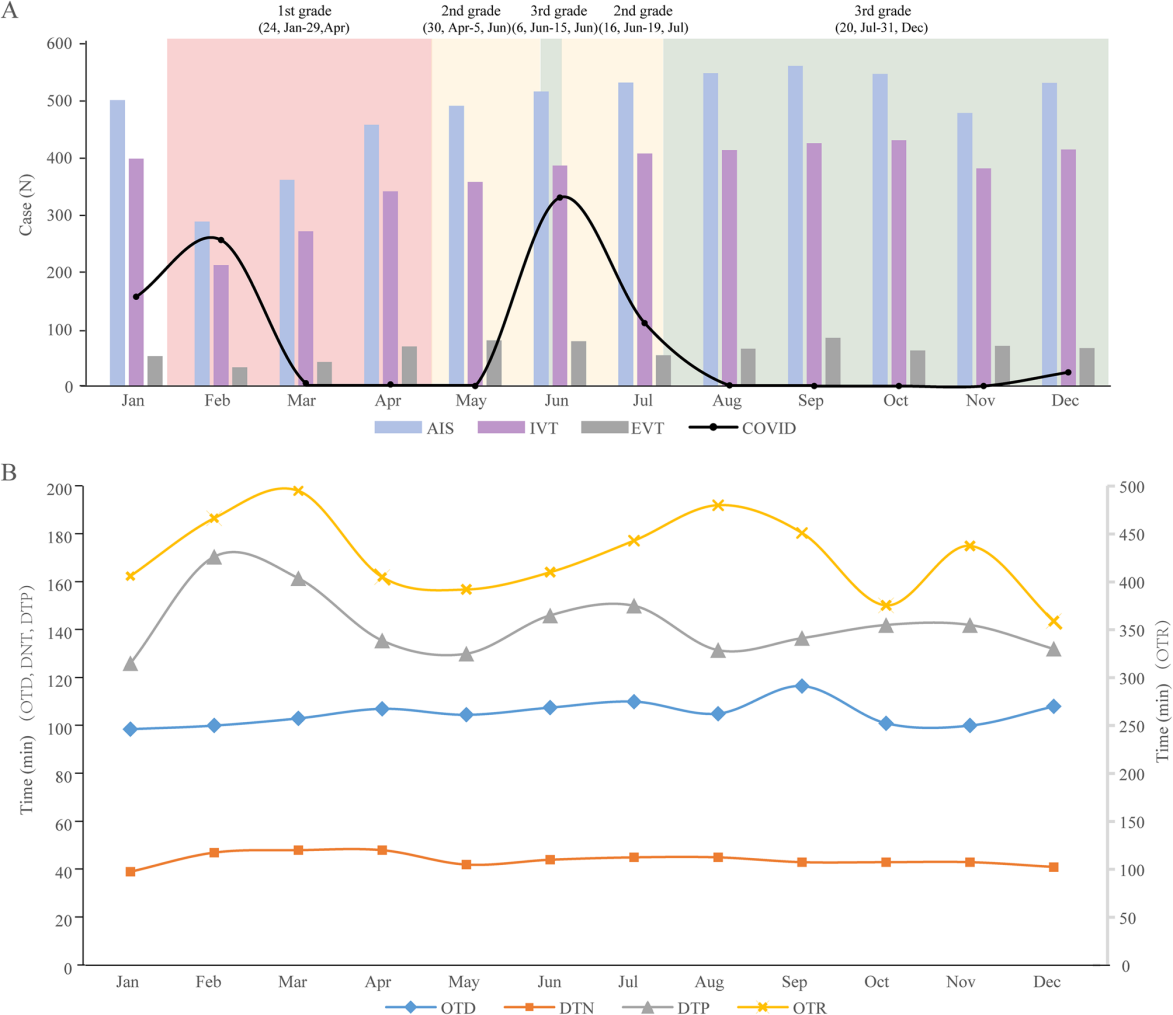
Monthly Quality Metrics for AIS in 2020. (**A**) Monthly volume of strokes across Beijing Stroke Quality Control Centre and COVID-19 cases in Beijing in 2020. (**B**) Mean timings of stroke activations by months from January 2020 to December 2020.

During the pandemic period, the number of stroke hospital admissions dropped significantly in February, increased from March, and it became steady from May. Compared to January 2020, the number of AIS patients declined by 42.5% in February 2020. When the patients were grouped according to monthly epoch, the door-to-treatment time during the COVID-19 period began to increase in February 2020 with persistent treatment delays in March and April 2020, which was in accordance with the lockdown period in Beijing (Fig. 3, Table 3).

**Table 3:** Quality measures of the acute care provided for ischemic stroke in 2020.

2020	Jan	Feb	Mar	Apr	May	Jun	Jul	Aug	Sep	Oct	Nov	Dec
Coronavirus disease-2019 (COVID-19) new cases^a^, n	156	255	5	3	0	329	110	1	0	0	0	24
All admission, n	576	337	416	501	551	554	571	613	612	605	523	582
Acute ischemic stroke (AIS), n	499	287	360	456	489	514	530	546	559	545	477	529
Intravenous thrombolysis (IVT), n	397	211	271	340	356	385	406	412	424	429	380	413
Endovascular treatment (EVT), n	52	33	43	69	80	78	54	65	84	62	70	66
Onset to door (OTD), min	98.5	100	103	107	104.5	107.5	110	105	116.5	101	100	108
Door-to-needle (DT), min	39	47	48	48	42	44	45	45	43	43	43	41
Door to puncture (DTP), min	126	170.5	161.5	135.5	130	146	150	131.5	136.5	143	142	132
Onset to recanalization (OTR), min	406	466.5	495	405	392	410	443	480	451	375.5	437.5	359

^a^Data was obtained from Beijing Municipal Health Commission.

## Discussion

Since the beginning of the outbreak, China has adopted unprecedented measures, including large-scale application of social isolation, closing borders, and nationwide lockdown at a great economic cost. Due to the limited resources, workforce shortages, strict infection control, physical distancing regulations and stay-at-home policies^2^, infected patients, as well as health care systems were profoundly affected by the COVID-19 crisis^16^, particularly patients with cardiovascular and cerebrovascular diseases. Given that stroke incidence can show day-to-day fluctuations and can be influenced by multiple factors^17^, such as weather and changes in air pollution, we conducted an analysis of the annual data in 2020 (during the pandemic) and 2019 (prepandemic). In this study, we reported the impact of the COVID-19 outbreak on stroke care in Beijing, China.

Although the study conducted by Zhao et al.^18^ included a larger number of hospitals in China, which showed a significant drop in admissions, thrombolysis, and thrombectomy for stroke patients during the COVID-19 outbreak, and no actual time for prehospital or in-hospital delays was ascertained. In the present study, we found that the effect of the pandemic on stroke care was not only in quantity but also in quality. The results showed the following: (1) there was a clear drop in the number of AIS patients in 2020, with an increased rate of IVT or EVT; (2) the delay mainly occurred after hospital arrival during the pandemic compared with the prepandemic period, especially in hospitals in the high-risk areas; and (3) the reduction in the number of AIS and delay in the time course mostly occurred in the first wave of the pandemic coinciding with the first 3 months of lockdown in Beijing.

Because of the burden of the COVID-19 pandemic, the capacities of emergency services were reduced, and access to acute stroke diagnosis and time-dependent therapies was limited and delayed. Several studies have shown differences in attention to AIS compared with prepandemic times. Most of them demonstrated a considerable decline in the number of acute strokes attended during the epidemic peak^19–21^. The reported decreases in stroke cases of all types ranged from 10 to 30%^22^. Hsiao et al. found that not only did stroke consultations decline by 39% in the 5 weeks after the announcement of statewide school and restaurant closures in Ohio, Kentucky, and Indiana, but reperfusion treatments also appeared to decline by 31%, specifically thrombolysis by 33%^23^. In our study, compared with those in 2019, both the numbers of patients presenting with AIS and haemorrhagic stroke decreased notably in the emergency departments in 2020. Fear of the contagion and the public recommendation to demand emergency assistance only if necessary have been considered crucial contributors to avoiding seeking urgent hospital care. Furthermore, regional lockdown policies may also play an important role^24^. The entry of AIS patients from the surrounding cities into Beijing became complicated and difficult. It is noteworthy that the proportion of AIS patients treated with IVT and EVT was increased. A Singapore study showed that the number of stroke activations decreased while the proportion receiving acute recanalization therapy remained stable in the Disease Outbreak Response System Condition (DORSCON) Orange^25^. A four-year comparative study from Israel reported that the number of patients admitted to the ED with suspected AIS declined by 41% compared to the parallel period in 2019, whereas an increase was noted in the number of patients diagnosed with AIS who underwent treatment, with the number of endovascular thrombectomy procedures increasing throughout the examined year^26^. Nguyen-Huynh et al. also found that the regional stroke alert and ischaemic stroke discharge volumes in northern California decreased significantly in the early COVID-19 pandemic, but there were more severe strokes and more large vessel occlusions^27^. The increased reperfusion therapy rate implied that both patients and medical staff correctly realized the significance of urgent assessment and treatment despite the context of COVID-19^28^. However, the increased rate of treatment was likely related to the deceased total number of AIS patients who were not eligible for IVT or EVT treatment, such as those with mild stroke symptoms or exceeding the therapeutic window^29–31^. Therefore, highlighting public awareness of stroke and centralized diversion to protected stroke centres are operational and paramount to avoid the tragedies of potentially treatable patients with stroke being denied appropriate treatment during this pandemic^32^.

Although it did not reach statistical significance, the OTD time showed a tendency of shortening during the pandemic, especially in the low/medium-risk areas, which might benefit from the better traffic situation. Thus, the delayed DTN, OTN, DTP and OTR times mainly reflected the prolonged time from hospital arrival to treatment, which might be related to the shortage of stroke team members, slowing down the evaluations and the precautionary workflow^22,33^. In a Canadian study, Katsanos et al. detected in-hospital delays for AIS treatment during the COVID-19 pandemic, which were related to delays from hospital presentation to the acquisition of cranial CT imaging^34^. The Society of Vascular and Interventional Neurology Multicentre Collaboration reported that patients treated during COVID-19 were at lower odds of receiving thrombolysis within 60 min of arrival, with a median delay in door-to-treatment time of 4 min^35^. According to the guidelines of the National Health Commission, hospitals changed their IVT and EVT procedures during the pandemic. All newly admitted patients, including emergency cases, needed strict COVID-19 screening. A travel history inquiry, chest CT scanning, complete blood count test, body temperature measurement and the ensuing multidisciplinary consultation were performed^36^. Although the screening was performed simultaneously with the evaluation of AIS, the subsequent treatment was still delayed. As a result, establishing a fast-track COVID-19 screening process, such as developing a rapid laboratory test, optimizing resource allocation, is highly needed^18^.

A discrepancy between the two areas was shown in that the delays mostly occurred in hospitals in high-risk areas, suggesting a milder impact in areas with fewer COVID-19 cases. In accordance with the emergency capacity of AIS, the Beijing Municipal Health Commission revealed and updated the first aid map of stroke in Beijing every year, which listed hospitals with IVT and/or DVT qualification. In every district, there was at least one hospital with AIS emergency capacity. Therefore, optimized prehospital triage, giving preference to the delivery of patients to nearby centres with AIS emergency management ability, would facilitate access to stroke care.

Beijing recorded its first COVID-19 case on January 20, 2020. On January 24, Beijing activated the highest level of public health emergency response to contain the outbreak, which lasted for 119 days (the lockdown period). Starting on April 30, the emergency response level was downgraded gradually as the novel coronavirus epidemic was mitigated. However, as a new cluster of local COVID-19 infections emerged from the Xinfadi market, Beijing faced a formidable challenge again and upgraded the prevention level of all communities from June 16 to July 19. The present study included stroke admissions as long as eight months after the lockdown, which showed the trend in stroke care in a longer period after a national lockdown. The reduction in the number of AIS and delay in the time course mostly occurred in the first wave of the pandemic, coinciding with the first 3 months of lockdown. Kansagra et al.^37^ reported a decline of approximately 39% in the number of patients who received evaluations for AIS in late March in the USA, which declared a national emergency over COVID-19 on 13 March. In April, the Expert Committee of Stroke Prevention and Treatment Project of the National Health Commission released “Expert consensus on management of stroke green channel in the setting of COVID-19”, which considered the management of patients during the chain of acute stroke care^38^. Although there was a rebound in the epidemic, the quality metrics of the AIS emergency care chain were relatively stable since April 2020, with a tendency towards gradual improvement compared with those in the first phase of the COVID-19 pandemic. A series of measures were adopted, such as the establishment of hospitals designated for COVID-19, designation of an isolated area for triage of suspected or confirmed patients with COVID-19, strict screening of COVID-19 and appropriate personal protective equipment to reduce the infection risk, standardized management, trained staff, and multidepartment collaboration to simplify the procedure as much as possible. It is notable that no health care personnel of the procedural teams developed symptoms consistent with COVID-19, and no patient who was admitted was diagnosed with COVID-19. It has been suggested that the adopted strategies for AIS management during the COVID-19 pandemic have been effective. In a time of much uncertainty, it is necessary to share professional experience among clinicians^39,40^. Professional protocols and consensus statements^41,42^ on how to protect stroke care pathways in the pandemic may help us to face the enormous challenges in difficult conditions.

This study also has several limitations. First, this is a retrospective analysis of medical records and depended on complete and accurate records. Second, this study did not include AIS patients who were not referred to the emergency department. Third, clinical characteristics and long-term outcomes were not analysed because of the limited variables we collected. Finally, we cannot rule out the effects of unexamined variables.

## Conclusion

There was a massive shift in health care resources during the COVID-19 pandemic, and cerebrovascular disease maintained a substantial burden. Despite a significant decrease in AIS admissions and a delay in the door-to-treatment time, the proportion of patients being treated did not decrease and even increased during the COVID-19 pandemic. The disruption of emergency stroke care mainly occurred in the first wave of the COVID-19 outbreak, and innovations in emergency stroke care systems seemed to be effective during this challenging time. These results might be helpful to professionals and health care providers in making decisions in new situations in the future.

COVID-19 is unlikely to be a short-term pandemic and will change our way of delivering stroke care for years to come. The world should be proactive in taking actions to meet these challenges. Further studies are needed to evaluate the potential unintended reasons and implications for patient consequences and health care systems. The stroke care models need to adapt to the world needs in this public health crisis.

## Data Availability

Detailed data are available from the corresponding author on reasonable request.
